# Anstieg depressiver Symptome bei Jugendlichen und jungen Erwachsenen während des ersten Lockdowns in Deutschland: Ergebnisse des Beziehungs- und Familienpanels pairfam

**DOI:** 10.1007/s00103-021-03451-5

**Published:** 2021-11-03

**Authors:** Elias Naumann, Ellen von den Driesch, Almut Schumann, Carolin Thönnissen

**Affiliations:** 1 Universität Mannheim, B6 30–32, 68131 Mannheim, Deutschland; 2 Bundesinstitut für Bevölkerungsforschung (BiB), Wiesbaden, Deutschland; 3 Wissenschaftszentrum Berlin für Sozialforschung (WZB), Berlin, Deutschland; 4 Institut für Soziologie und Sozialpsychologie (ISS), Universität Köln, Köln, Deutschland

**Keywords:** COVID-19, Psychische Gesundheit, Ungleichheit, Geschlechterunterschiede, Migrationshintergrund, COVID-19, Mental health, Inequality, Gender differences, Migration

## Abstract

**Hintergrund:**

Die COVID-19-Pandemie (Corona Virus Disease 2019) hat innerhalb kürzester Zeit das gesellschaftliche Leben grundlegend verändert. Bei politischen Entscheidungen steht oft die Abwägung zwischen der Pandemiebekämpfung und den möglichen negativen wirtschaftlichen Konsequenzen im Vordergrund. Zunehmend finden jedoch auch die psychologischen und sozialen Auswirkungen des Lockdowns Beachtung.

**Fragestellung:**

Wie hat sich die psychische Gesundheit von Jugendlichen und jungen Erwachsenen in Deutschland während der ersten Welle der COVID-19-Pandemie und der dadurch bedingten Kontaktbeschränkungen in Deutschland verändert?

**Material und Methoden:**

Die Analysen basieren auf Längsschnittdaten von bundesweit per Zufallsverfahren ausgewählten Ankerpersonen des Beziehungs- und Familienpanels pairfam. Die hier betrachtete Altersgruppe der Geburtsjahrgänge 2001–2003 wurde im Jahr 2018/2019 erstmalig im Zuge einer Aufstockungsstichprobe befragt und 854 dieser Jugendlichen und jungen Erwachsenen im Alter von 16–19 Jahren nahmen auch an der COVID-19-Zusatzbefragung von Mai bis Juli 2020 (erster Lockdown) teil. Die Depressivität wird mit der State-Trait Depression Scale erhoben.

**Ergebnisse:**

Während des ersten Lockdowns zeigte sich bei den jungen Menschen ein deutlicher Anstieg depressiver Symptome: Vor dem Lockdown hatten 10,4 % klinisch relevante depressive Symptome [95 %-KI: 8,4; 12,5], im Frühjahr 2020 stieg dieser Anteil auf 25,3 % [95 %-KI: 22,4; 28,2]. Das Risiko, depressive Symptome zu entwickeln, war bei weiblichen Jugendlichen und jungen Frauen erhöht. Der Migrationshintergrund zeigte sich als ein ähnlich starker Risikofaktor: Die Prävalenz depressiver Symptome stieg bei Migrationshintergrund von 11 % auf 33 %.

**Diskussion:**

Um diese Risikogruppen zu erreichen, sind flächendeckende, zielgruppenspezifische und niedrigschwellige Angebote der Prävention und Gesundheitsförderung nötig.

## Abstract (en)

**Background:**

The COVID-19 pandemic has fundamentally changed social life within a very short time. Lockdown policies often consider the tradeoff between containing the spread of the pandemic and negative consequences for the economy. Policymakers should pay more attention to the psychological and social impacts of the lockdown.

**Research question:**

How did the mental health of adolescents in Germany change during the first wave of the COVID-19 pandemic and the lockdown?

**Materials and methods:**

Analyses are based on longitudinal data from nationwide randomly selected anchors of the German family panel pairfam. The age group considered here, born between 2001 and 2003, was surveyed for the first time in 2018/2019 in the course of a refreshment sample, and 854 of these adolescents and young adults aged 16–19 also participated in the COVID-19 supplementary survey from May to July 2020 (first lockdown). Depressiveness is assessed with the State-Trait Depression Scale.

**Results:**

During the first lockdown, adolescents show a significant increase in depressive symptoms. Prior to the lockdown, 10.4% had clinically relevant depressive symptoms [95% CI: 8.4; 12.5]. In spring 2020, the prevalence increased to 25.3% [95% CI: 22.4; 28.2]. Young women have a significantly higher risk of developing depressive symptoms than men of the same age. Immigrant background is an equally strong risk factor. The prevalence of depressive symptoms among adolescents with an immigrant background increased from 11% to 33%.

**Discussion:**

To address this increased mental health risk and the inequalities, policymakers and society should ensure access and availability of target-group-specific and low-threshold prevention and counselling.

## Einleitung

Die COVID-19-Pandemie hat innerhalb kürzester Zeit das gesellschaftliche Leben grundlegend verändert. Dabei steht bei politischen Entscheidungen oft die Abwägung zwischen der Pandemiebekämpfung und den möglichen negativen wirtschaftlichen Auswirkungen im Vordergrund. Zunehmend finden jedoch auch die psychologischen und sozialen Auswirkungen des Lockdowns Aufmerksamkeit. In diesem Artikel untersuchen wir, wie sich die psychische Gesundheit von Jugendlichen in Deutschland während der ersten Welle der COVID-19-Pandemie und der dadurch bedingten Kontaktbeschränkungen in Deutschland verändert hat.

Das tägliche Leben von Kindern und Jugendlichen hat sich seit März 2020 massiv verändert. Schulschließungen führen zum Wegfall vieler sozialer Kontakte und Regeln zur sozialen Distanzierung schränken den Kontakt auch außerhalb der Schule ein. Vereinsaktivitäten, Freizeitangebote und Sport fallen größtenteils weg, die Bildschirmzeit nimmt zu. Dabei gelten sowohl (sportliche) Aktivität als auch soziale Kontakte als wichtiger Schutz vor psychischen Erkrankungen, insbesondere Depressionen. Bisherige Forschung zeigt, dass diese Veränderungen Kinder und Jugendliche erheblich belasten können [1, 2]. Gleichzeitig waren in vielen Fällen aber auch die Möglichkeiten der Eltern begrenzt, ihre Kinder in dieser Zeit zu begleiten und zu unterstützen. Viele Eltern sind selbst vor die Herausforderung gestellt, Arbeit und Kinderbetreuung zu vereinbaren, und manche haben zudem gesundheitliche oder auch finanzielle Sorgen aufgrund der COVID-19-Pandemie [3].

Insgesamt ist also zu erwarten, dass die COVID-19-Pandemie und die damit verbundenen Lockdownmaßnahmen bei Kindern und Jugendlichen zu psychosozialem Stress führen. Gleichzeitig sind Resilienzfaktoren wie Kontakte zu Freunden oder eine gute Beziehung zu den Eltern [4] nur eingeschränkt vorhanden, sodass letztlich eine Zunahme psychischer Probleme zu erwarten ist. Eine Abschätzung des Ausmaßes dieser Zunahme ist bisher jedoch unbekannt – genauso wie die Identifizierung von Risikogruppen, die besonders stark durch die COVID-19-Pandemie beeinflusst sind.

Bestehende Studien zur psychischen Gesundheit von Erwachsenen zeigen Anstiege bei Depressionen und Ängsten im Vergleich zur Zeit vor der COVID-19-Pandemie (z. B. [5–9]). Skoda et al. [7] berichten von einer deutlichen Zunahme depressiver Symptome, deren Prävalenz von 8 % auf 21 % zunimmt (gemessen mit dem Patient Health Questionnaire-2). Peters et al. [10] hingegen finden, dass die Prävalenz moderater bis schwer ausgeprägter depressiver Symptome von 6,4 % auf 8,8 % steigt, allerdings findet sich diese Zunahme depressions- und angstassoziierter Symptome im Vergleich zur letzten Untersuchung vor COVID-19 nur bei Teilnehmenden unter 60 Jahren, insbesondere bei jungen Frauen.

Verlässliche Ergebnisse zur psychischen Gesundheit von Kindern während der COVID-19-Pandemie gibt es bisher wenig. Eine Ausnahme ist die Studie von Ravens-Sieberer et al. [11], die ein repräsentatives Sample von Kindern und Jugendlichen im Alter von 7 bis 17 Jahren in Deutschland untersucht. Vergleiche mit Daten aus der Zeit vor COVID-19 zeigen eine signifikante Abnahme der gesundheitsbezogenen Lebensqualität, mehr psychische Probleme (17,8 % vs. 9,9 %) und höhere Angstwerte (24,1 % vs. 14,9 %) als vor der Pandemie. Überraschenderweise zeigt sich jedoch keine Zunahme depressiver Symptome bei den Kindern (siehe auch [12]).

Der Beitrag unserer Studie liegt daher darin, dass mit den 16- bis 19-jährigen Jugendlichen eine Gruppe in den Blick genommen wird, zu der bisher noch keine Ergebnisse vorliegen. Das liegt womöglich unter anderem daran, dass sie sowohl in den Erwachsenenstudien als auch in den Kinderstudien keine Berücksichtigung finden. Zudem ist der Fokus auf depressive Symptome bei Jugendlichen deshalb sehr relevant, weil das Auftreten depressiver Symptome im Jugendalter von Komorbiditäten – wie Gewichts- und Appetitstörungen, Schlafstörungen, motorische Störungen, Denkschwierigkeiten und im Extremfall Suizid – begleitet sein kann und das Risiko psychischer Erkrankungen im Lebensverlauf erhöht [13].

Es wird zunächst die zugrunde liegende Datenbasis dargelegt und erläutert, welche Messungen wir zur Diagnostik der Depressivität aus den Daten des Beziehungs- und Familienpanels pairfam in unseren Analysen verwenden. Anschließend werden die Befunde im Ergebnisteil präsentiert, gefolgt von einer Einordnung der Robustheit und möglicher Limitationen der Daten und Methodik. Auf Basis der Ergebnisse werden abschließend einige Handlungsempfehlungen für politische Maßnahmen diskutiert.

## Material und Methoden

Die Analysen basieren auf längsschnittlichen Daten des Beziehungs- und Familienpanels pairfam [14] und der online durchgeführten pairfam-COVID-19-Zusatzbefragung [15]. Bei den pairfam-Daten handelt es sich um eine zufallsbasierte Registerstichprobe. In diesem Artikel konzentrieren wir uns auf Befragte der Jugendlichenkohorte der Geburtsjahrgänge 2001–2003, die also im ersten Befragungszeitraum von November 2018 bis Ende Juli 2019 15–17 Jahre alt waren. An der Befragung im Jahr 2018/2019 haben insgesamt 2465 Personen im entsprechenden Alter teilgenommen, mit einer Ausschöpfung von 28,9 % der gezogenen Registerdaten. An der COVID-19-Zusatzbefragung nahmen zwischen dem 19.05. und dem 13.07.2020 854 Jugendliche (bzw. inzwischen teilweise junge Erwachsene) teil, sodass 34,6 % der ursprünglich Befragten wiederbefragt werden konnten.

Für alle Analysen wird in der vorliegenden Studie durchgehend ein kalibriertes Designgewicht verwendet, welches zum einen die Verteilung der Panelstichprobe an die Zielpopulation, vor allem in Hinblick auf Geschlecht und Wohnort, angleicht und zum anderen die unterschiedlichen Teilnahmebereitschaften in der COVID-19-Zusatzbefragung kontrolliert. Somit kann eine mögliche Verzerrung aufgrund von Panelmortalität ausgeglichen werden [15]. Für die verwendete Stichprobe zeigen sich nur leichte Abweichungen: Während Mädchen und junge Frauen eher an der Wiederbefragung teilgenommen haben als Jungen und junge Männer, hat der Wohnort kaum einen Einfluss auf die Wahrscheinlichkeit, an der Wiederbefragung teilzunehmen (vgl. Zusammensetzung der Stichprobe in Tab. 1). Die Randverteilungen der gewichteten Stichprobe entsprechen denen der Zielpopulation, sodass die wiederbefragten Personen repräsentativ für die Zielpopulation sein sollten.

**Table 1:** 

	Befragung 2018/2019 (ungewichtet) *n* = 2465 (in %)	Befragung 2018/2019 (gewichtet) (in %)	COVID-19-Zusatzbefragung (ungewichtet) *n* = 854 (in %)	COVID-19-Zusatzbefragung (gewichtet) (in %)
*Geschlecht*				
Männlich	50,9	51,2	42,3	51,2
Weiblich	49,1	48,8	57,7	48,8
*Wohnort Bundesland*				
Baden-Württemberg	12,7	14,8	14,1	14,3
Bayern	19,2	15,6	22,0	15,9
Berlin (Ost)	2,2	1,9	2,2	1,7
Berlin (West)	2,0	1,6	1,9	1,5
Brandenburg	2,4	2,8	2,3	2,7
Bremen	0,9	0,7	0,5	0,9
Hamburg	2,0	1,9	1,6	2,1
Mecklenburg-Vorpommern	1,7	1,5	2,0	1,5
Niedersachsen	10,5	9,8	9,5	10,2
Nordrhein-Westfalen	18,4	23,0	16,0	22,5
Hessen	9,9	8,0	10,7	8,2
Rheinland-Pfalz	4,7	4,9	4,8	4,8
Saarland	1,5	1,2	1,3	1,1
Sachsen	3,5	3,9	3,4	4,0
Sachsen-Anhalt	2,0	2,2	1,9	2,2
Schleswig-Holstein	3,8	3,9	3,9	3,9
Thüringen	2,6	2,4	2,1	2,4

Depressivität wird entsprechend der deutschen Adaption der State-Trait Depression Scale (STDS, [16]) gemessen. Mit insgesamt 10 Items konnten die Befragten angeben, wie häufig sie verschiedene Gefühle, wie zum Beispiel Glück, Verzweiflung oder Schwermut, während der Zeit des ersten Lockdowns in Deutschland erlebt haben. Aus diesen Items wird ein Summenindex gebildet. Skalenwerte von mehr als 25 Punkten gelten als Hinweis auf klinisch relevante depressive Symptome [17]. Die STDS wurde auch in der pairfam-Welle im Jahr 2018/2019 erhoben und weist zu beiden Zeitpunkten eine hohe Reliabilität auf (Cronbachs Alpha: 0,86 (2018/2019) bzw. 0,90 (2020)). Da die Zuverlässigkeit der Messungen der Depressivitätsskala, gemessen an Cronbachs Alpha, zu beiden untersuchten Zeitpunkten ähnlich hoch ist, ist ein Vergleich dieser Werte vor und während der Coronakrise bei den Befragten möglich.

Standardisierte diagnostische Interviews, wie z. B. das Composite International Diagnostic Interview (CIDI), gelten als Goldstandardinstrumente zur Bestimmung der Prävalenz von Depressionen. Das Munich-Composite International Diagnostic Interview (M-CIDI) weist relativ zu einer klinischen Diagnose für einfache bzw. wiederkehrende depressive Episoden eine Sensitivität von 92 % und eine Spezifität von 100 % auf [18]. Um das Vorhandensein depressiver Symptome und ihren Schweregrad in Umfragen zu erfassen, werden verschiedene sogenannte Depressionsskalen eingesetzt. Hierzu gehören auf der Ebene der Selbstbeurteilungsverfahren (Patientenbeurteilung) u. a. die Center of Epidemiological Studies Depression Scale (CES-D), das Beck Depression Inventory (BDI) oder der Gesundheitsfragebogen für Patienten (PHQ‑D; [19]). Mit dem PHQ‑D wird eine Sensitivität für eine depressive Episode von 77–88 % und eine Spezifität von 88–94 % erreicht [20]. Der CES‑D hat eine Sensitivität von 89 % und eine Spezifizität bei 86 %. Die von uns verwendete STDS weist eine Sensitivität von 76 % und eine Spezifizität von 93,3 % auf und stellt damit ein Screeningverfahren zur Verfügung, das hoch reliabel und ökonomisch ein überdurchschnittliches bis klinisch hoch bedeutsames Ausmaß an Depressivität erfasst. „Ihre Anwendung in epidemiologischen Studien zur Identifikation depressiver Störungen kann empfohlen werden“ [17].

Die stärksten Risikofaktoren für Depressionen bei Jugendlichen sind eine Depressionsanamnese in der Familie, Entwicklungsfaktoren, Sexualhormone und die Belastung durch psychosozialen Stress [13]. Demnach haben Mädchen ein höheres Risiko an Depressionen zu leiden. Zudem zeigt sich ein Zusammenhang zwischen Depressionen und Umweltfaktoren wie akuten Stressereignissen (z. B. Verletzungen, Trauerfälle) und anderen Widrigkeiten (z. B. familiäre Unstimmigkeiten, Mobbing durch Gleichaltrige, Armut, körperliche Krankheit). Schließlich gelten gute zwischenmenschliche Beziehungen als Schutz gegen Depressionen [13].

**Table 2:** 

–	*n*	Anteile (ungewichtet) (in %)
*Geschlecht*		
Weiblich	388	57,3
Männlich	289	42,7
*Migrationshintergrund*		
Nein	540	79,8
Ja (erste oder zweite Generation)	137	20,2
*Aktuelle Erwerbssituation (in 2020)*		
Schüler/in	556	82,1
Ausbildung Lehre	85	12,6
Studium	36	5,3
*Bildung der Mutter (ISCED 97)*		
Niedrig (vorschulische Bildung, Grundbildung und Sekundarbildung der Unterstufe (Level 0 bis 2))	33	4,9
Mittel (Sekundarbildung Oberstufe (Hochschulzugangsberechtigung) und postsekundäre, nichttertiäre Bildung (Berufsausbildung o. Ä.; Level 3 und 4))	376	55,5
Hoch (erste und zweite Stufe der tertiären Bildung (d. h. mind. Hochschulabschluss; Level 5 und 6))	268	39,6
*Finanzielle Situation *(„Wir müssen häufig verzichten, wegen finanzieller Einschränkungen“)		
1 Stimme überhaupt nicht zu	376	55,5
2	207	30,6
**3**	65	9,6
4	21	3,1
5 Stimme voll und ganz zu	8	1,2
*Wohnform*		
Bei Eltern (ohne Geschwister)	208	30,7
Bei Eltern (mit Geschwistern)	448	66,2
Nicht mehr bei den Eltern	21	3,1
*Wohnort*		
Stadt	437	64,6
Land	240	35,4

*n* = 677: Befragte, die in die multivariate Analyse eingehen (Tab. 3), d. h. diejenigen, die in 2018/2019 keine depressiven Symptome und gültige Werte für alle Kontrollvariablen hatten

Ausgehend von diesen Befunden untersuchen wir, welche individuellen Eigenschaften und Lebensumstände mit dem Auftreten von Depressionen zusammenhängen. Als unabhängige Variablen verwenden wir daher das Geschlecht, den Migrationshintergrund der Jugendlichen und ihren Erwerbsstatus. Ein Indikator für die soziale Einbettung ist, ob die Jugendlichen bei den Eltern mit oder ohne Geschwister oder alleine leben. Zusätzlich wird der Bildungshintergrund über den Bildungsstand der Mutter erhoben. Wir verwenden den höchsten Schulabschluss (International Standard Classification of Education, ISCED 97) und unterscheiden niedrige Bildung (Level 0–2: vorschulische Bildung, Grundbildung und Sekundarbildung der Unterstufe), mittlere Bildung (Level 3 und 4: Sekundarbildung Oberstufe [Hochschulzugangsberechtigung] und postsekundäre, nichttertiäre Bildung [Berufsausbildung o. Ä.]) und hohe Bildung (Level 5 und 6: erste und zweite Stufe der tertiären Bildung [d. h. mind. Hochschulabschluss]). Zur Erfassung der ökonomischen Deprivation verwenden wir die subjektive Einschätzung der finanziellen Situation des Haushalts („Wir müssen häufig auf etwas verzichten, weil wir uns finanziell einschränken müssen“). Die Häufigkeitsverteilungen der unabhängigen Variablen finden sich in Tab. 2.

## Ergebnisse

**Figure 1:**
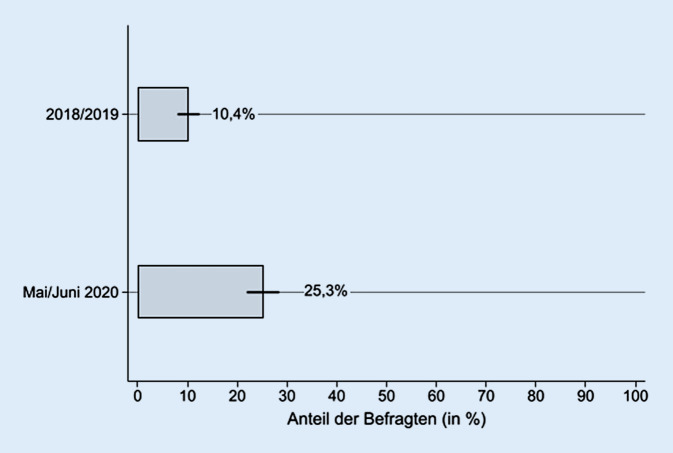

Während des ersten Lockdowns zeigte sich bei den Jugendlichen und jungen Erwachsenen ein deutlicher Anstieg depressiver Symptome. Vor dem Lockdown, im Jahr 2018/2019, hatten 10,4 % der jungen Menschen klinisch relevante depressive Symptome (95 %-KI: 8,4; 12,5). Das entspricht Schätzungen zur Prävalenz von depressiven Symptomen aus anderen Studien [21]. Im Frühjahr 2020 stieg dieser Anteil auf 25,3 % an (95 %-KI: 22,4; 28,2; Abb. 1). Jeder vierte junge Mensch zwischen 16 und 19 Jahren wies dementsprechend klinisch relevante Symptome einer Depression auf. Das ist eine statistisch signifikante Zunahme um 14,9 Prozentpunkte (95 %-KI: 11,8; 18,0).

Nicht alle jungen Menschen waren jedoch in gleicher Weise von den negativen Auswirkungen der Kontaktbeschränkungen betroffen. Zudem unterscheiden sie sich in den Möglichkeiten, mit den Einschränkungen des Lockdowns umzugehen. Um diese Gruppen besser identifizieren zu können, unterscheiden wir im Folgenden diejenigen Jugendlichen und jungen Erwachsenen, die bei der Befragung im Jahr 2018/2019 keine klinisch relevanten depressiven Symptome aufwiesen (*n* = 765), von denjenigen, die bereits 2018/2019 klinische Symptome gezeigt haben (*n* = 89; 10,4 %). Von Letzteren hatten fast 60 % auch im Frühjahr 2020 noch erhöhte Werte auf der Depressivitätsskala, während bei 40 % (*n* = 36) der Befragten der Wert wieder unter den Grenzwert gefallen ist.

Zwischen den beiden Befragungen entwickelten 21,3 % (*n* = 163) der Jugendlichen und jungen Erwachsenen klinisch relevante depressive Symptome. Mit einer logistischen Regression schätzen wir im Folgenden, welche Jugendlichen ein erhöhtes Risiko haben, depressive Symptome zu entwickeln. Eine Übersicht der verwendeten unabhängigen Variablen und ihrer Codierungen findet sich in Tab. 2.

**Table 3:** 

–	Auftreten depressiver Symptome
*Geschlecht:* weiblich	2,767^b^
–	(1,772; 4,321)
*Migrationshintergrund*	1,793^a^
–	(1,064; 3,022)
*Erwerbsstatus *(Ref.-Kat.: Schüler/in)	
in Ausbildung	0,852
–	(0,458; 1,586)
Studierende	1,182
–	(0,422; 3,313)
*Bildungsstand* der Mutter ISCED 97 (Ref.-Kat.: mittel)	
Niedrig	0,941
–	(0,381; 2,326)
Hoch	0.761
–	(0,485; 1,194)
*Finanzielle Situation des Haushaltes*	0.992
–	(0,791; 1,245)
*Wohnform* (Ref.-Kat.: bei den Eltern ohne Geschwister)	
Bei Eltern (mit Geschwistern)	1.995^b^
–	(1,204; 3,308)
Nicht bei den Eltern	4.622^b^
–	(1,676; 12,75)
*Wohnort:* Land	0.964
–	(0,616; 1,510)
Pseudo‑R^2^	0,07
*n*	677

Odds Ratios; 95 %-Konfidenzintervalle in Klammern

^a^*p* < 0,05

^b^*p* < 0,01, Balancierte Stichprobe (*n* = 677 zu beiden Zeitpunkten)

Weibliche Jugendliche und junge Erwachsene hatten eine höhere Wahrscheinlichkeit, depressive Symptome zu entwickeln, als männliche im gleichen Alter. Während sich bei Jungen und jungen Männern die Prävalenz depressiver Symptome von 7 % auf 15 % erhöhte, war der Anstieg bei den Mädchen und jungen Frauen deutlich stärker (von 13 % auf 35 %). Der Geschlechterunterschied in der Prävalenz von Depressionen vor der Pandemie lag also bei 2:1 (weiblich:männlich), was den Ergebnissen bisheriger Forschung entspricht [21]. Dieses Verhältnis ist während der Pandemie auf 3:1 angestiegen. Dies bestätigt sich auch in der multivariaten Auswertung (Tab. 3). Das Chancenverhältnis, dass weibliche im Vergleich zu männlichen jungen Menschen depressive Symptome entwickeln, erhöhte sich hier um den Faktor 2,8 (95 %-KI: 1,7; 4,3).

Der Migrationshintergrund scheint ein ähnlich starker Risikofaktor zu sein. Jugendliche und junge Erwachsene mit Migrationshintergrund zeigten im Vergleich zu jenen ohne Migrationshintergrund eine höhere Wahrscheinlichkeit depressive Symptome zu entwickeln. Während bei den jungen Menschen ohne Migrationshintergrund der Anteil mit depressiven Symptomen von 9 % auf 21 % anstieg, verdreifachte sich der Anteil derjenigen mit Migrationshintergrund von 11 % auf 33 %.

Das Bildungsniveau der Eltern – unabhängig davon, ob man das Bildungsniveau der Mutter oder des Vaters nimmt und ob die Befragten Schüler, Studierende oder Auszubildende sind – hatte hingegen keinen Einfluss auf die Wahrscheinlichkeit, depressive Symptome während des Lockdowns zu entwickeln. Auch zwischen der finanziellen Situation des Haushaltes und dem Auftreten von depressiven Symptomen zeigt sich kein Zusammenhang.

Einen starken Einfluss hat jedoch, ob die Befragten bei den Eltern wohnen: Die Wahrscheinlichkeit, depressive Symptome zu entwickeln, war deutlich geringer, wenn die Befragten noch bei den Eltern wohnen im Vergleich zu denen, welche das Elternhaus verlassen haben. Vergleicht man Jugendliche und junge Erwachsene, die noch bei den Eltern wohnen, so zeigt sich überraschenderweise, dass das Vorhandensein von Geschwistern mit einem höheren Risiko depressiver Symptome zusammenhängt.

## Diskussion

Bei Jugendlichen hat sich die psychische Gesundheit unter den Bedingungen der COVID-19-Pandemie und den Eindämmungsmaßnahmen deutlich verschlechtert und depressive Symptome haben im Vergleich zur Zeit vor der Pandemie deutlich zugenommen (siehe auch [11, 12]). Dies gilt insbesondere für Mädchen und junge Frauen sowie für junge Menschen mit Migrationshintergrund. Sie gehören zu den Gruppen in der Bevölkerung, bei denen sich ein besonders starker Zusammenhang zwischen den Kontaktbeschränkungen und depressiven Symptomen zeigt und die somit einem besonderen Risiko ausgesetzt sind. Der Unterschied zwischen den Prävalenzen bei beiden Geschlechtern ist nicht überraschend. Sie nimmt mit steigendem Alter zu [22, 23]. Während weibliche Jugendliche und junge Erwachsene vermehrt Symptome von Depressivität und Angst zeigen, also internalisierendes Verhalten, kommt es bei den männlichen verstärkt zu externalisierendem Verhalten, wie Problemen im Sozialverhalten oder Aufmerksamkeitsdefizit- und Hyperaktivitätsstörung (ADHS; [24]). Weitere Forschung sollte demnach gezielt schauen, ob diesen strukturellen Ungleichheiten, beruhend vor allem auf dem Geschlecht und dem Migrationshintergrund, weitere erklärende Mechanismen zugrunde liegen. Eine adäquate Ursachenforschung in diesen Gruppen kann schließlich helfen, diese Ungleichheiten zu verringern und flächendeckende, zielgruppenspezifische und niedrigschwellige Angebote der Prävention und Gesundheitsförderung auszubauen und zu fördern.

Erst langsam wächst ein Bewusstsein hierfür. Angesichts dieser Erkenntnis sollten politische Maßnahmen gezielt auch diese Personengruppen unterstützen. Ein Ausbau der psychologischen Beratungsstellen, Familienbildungsstätten und ambulanten sowie stationären Therapieplätze wäre eine Möglichkeit, die Risikogruppen zu begleiten. Ebenso ist der Ausbau niederschwelliger, digitaler Hilfsangebote sicherlich eine Maßnahme, mit der man junge Menschen gut direkt erreichen könnte. Und schließlich können sicherlich auch Eltern noch besser dabei unterstützt werden, ihre Kinder im Umgang mit den Einschränkungen zu begleiten [27].

Auf Basis unserer Daten kann jedoch nicht eindeutig geklärt werden, ob für den Anstieg der Depressionssymptomatik bei Jugendlichen und jungen Erwachsenen allein der Lockdown und die damit einhergehenden Eindämmungsmaßnahmen verantwortlich sind. Vielmehr können auch weitere indirekte Folgen der Pandemie, beispielsweise im gesundheitlichen oder ökonomischen Familienumfeld, einen Einfluss auf die mentale Verfassung der jungen Generation haben. Zudem ist denkbar, dass dem Auftreten von Depressionen übliche Alterseffekte zugrunde liegen.

### Robustheitsprüfung

Die Ergebnisse unserer Analysen basieren auf der Befragung des Familienpanels pairfam 2018/2019 und könnten aufgrund von Panelmortalität verzerrt sein. Daher sind alle unsere Ergebnisse gewichtet. Bezüglich der Teilnahmebereitschaft an der COVID-19-Zusatzbefragung können wir zeigen, dass diese nicht mit den depressiven Symptomen in der ersten Befragung zusammenhängt. Befragte mit depressiven Symptomen in der ersten Befragung haben also eine ähnliche Wahrscheinlichkeit, an der COVID-19-Zusatzbefragung teilzunehmen, wie Befragte ohne depressive Symptome. Dennoch ist denkbar, dass die Teilnahmebereitschaft bei der Zusatzbefragung mit dem Entstehen von depressiven Symptomen zusammenhängt. Das wäre dann der Fall, wenn diejenigen, die zwischen der ersten und der Zusatzbefragung depressive Symptome entwickeln, nicht mehr an der Zusatzbefragung teilnehmen. Forschung zu solchen Selektionseffekten zeigt jedoch, dass Befragte mit einer guten Gesundheit und einem guten psychischen Wohlbefinden eher an Folgestudien teilnehmen [25]. Das würde heißen, dass wir den Effekt des Lockdowns auf die psychische Gesundheit eher unter- als überschätzen.

Mode-Effekte sind eine zweite mögliche Ursache, weshalb die Ergebnisse verzerrt sein könnten. Unter Mode-Effekten werden Verzerrungen in den Antworten von Befragten verstanden, die auf die Form und Art der Durchführung der Befragung zurückzuführen sind. Die Befragungen der Erhebung 2018/2019 erfolgten anhand persönlicher Interviews (CAPI). Bestimmte sensitive Fragen, wie die von uns verwendeten STDS-Fragen, wurden jedoch im selbstadministrierten computergestützten Interview (CASI) abgefragt, bei dem die Teilnehmenden die Antworten selbstständig am Computer ausfüllen. Da während des COVID-19-Lockdowns persönliche Interviews im Haushalt der Befragten nicht möglich waren, wurde die Zusatzbefragung im Jahr 2020 mithilfe von selbstadministrierten Webinterviews (CAWI) durchgeführt. Die Befragungssituation ist hierbei durch eine höhere Anonymität gekennzeichnet und beugt allgemeinhin sozial erwünschtem Antwortverhalten vor. Mögliche Effekte aufgrund des Wechsels des Befragungsmodus zwischen beiden Befragungen sollten jedoch aufgrund des CASI-Einsatzes eine untergeordnete Rolle spielen, da der Befragte in beiden Interviewsituationen die Fragen selbst administriert beantworten konnte, ohne dass ein Interviewer Einblick in das Antwortverhalten hatte. Dennoch ist nicht ganz auszuschließen, dass die Anwesenheit eines Interviewers in der ersten Befragung womöglich zu einer Verzerrung des Antwortverhaltens in Richtung sozialer Erwünschtheit geführt haben könnte.

Saisonale Schwankungen von depressiven Symptomen sind eine dritte Möglichkeit, weshalb unsere Ergebnisse verzerrt sein könnten. Forschungsergebnisse dazu sind bisher nicht eindeutig, legen jedoch nahe, dass depressive Symptome im Winter häufiger auftreten [26]. In unsere Analysen der ersten Befragung flossen zu etwa 50 % Interviews ein, die zwischen Dezember 2018 und März 2019, also in den Wintermonaten, stattgefunden hatten, während sämtliche Interviews der COVID-19-Zusatzbefragung im Mai und Juni 2020 erfolgten. Wir können somit nicht ausschließen, dass depressive Symptome auch bei unseren Befragten im Frühjahr seltener sind als im Winter. Das hieße aber auch, dass wir den Anstieg der depressiven Symptome eher unter- als überschätzen.

## Fazit

Nach über einem Jahr der COVID-19-Pandemie mit mehreren Lockdowns und wiederholten Phasen von Schulschließungen machen unsere Befunde deutlich, dass die psychische Gesundheit von Jugendlichen und jungen Erwachsenen bei zukünftigen politischen Entscheidungen unbedingt stärker beachtet werden sollte. Dazu gehört, dass Politik und Gesellschaft Wege finden, die psychische Gesundheit von jungen Menschen zu schützen und aufrechtzuerhalten und besonders belastete Jugendliche und deren Eltern zu unterstützen.
